# Network estimation for censored time-to-event data for multiple events based on multivariate survival analysis

**DOI:** 10.1371/journal.pone.0239760

**Published:** 2020-10-01

**Authors:** Yoojoong Kim, Junhee Seok

**Affiliations:** School of Electrical Engineering, Korea University, Seoul, South Korea; Universiteit Maastricht, NETHERLANDS

## Abstract

In general survival analysis, multiple studies have considered a single failure time corresponding to the time to the event of interest or to the occurrence of multiple events under the assumption that each event is independent. However, in real-world events, one event may impact others. Essentially, the potential structure of the occurrence of multiple events can be observed in several survival datasets. The interrelations between the times to the occurrences of events are immensely challenging to analyze because of the presence of censoring. Censoring commonly arises in longitudinal studies in which some events are often not observed for some of the subjects within the duration of research. Although this problem presents the obstacle of distortion caused by censoring, the advanced multivariate survival analysis methods that handle multiple events with censoring make it possible to measure a bivariate probability density function for a pair of events. Considering this improvement, this paper proposes a method called censored network estimation to discover partially correlated relationships and construct the corresponding network composed of edges representing non-zero partial correlations on multiple censored events. To demonstrate its superior performance compared to conventional methods, the selecting power for the partially correlated events was evaluated in two types of networks with iterative simulation experiments. Additionally, the correlation structure was investigated on the electronic health records dataset of the times to the first diagnosis for newborn babies in South Korea. The results show significantly improved performance as compared to edge measurement with competitive methods and reliability in terms of the interrelations of real-life diseases.

## Introduction

With the recent emergence of large-scale and complex data, it is important to uncover relationships among variables during data analyses. There has been a large amount of research inferring the interdependency of variables from the data and presenting networks in which nodes and edges correspond to the variables and their relations, respectively. The edges in the network can be interpreted as a conditional dependency corresponding to non-zero entries of the inverse covariance matrix [1]. This work is utilized to elucidate an underlying structure for variables in areas such as molecular network analysis in biomedicine [2] and social network analysis [3].

In terms of graphical models, several approaches have been proposed to estimate the partial correlations of variables in data. For multivariate data under the normality assumption, the inverse covariance matrix can be measured by neighborhood selection with the lasso [4], maximizing the Gaussian log-likelihood with block-wise coordinate descent and L1 regularization (Graphical Lasso) [5, 6], as well as sparse partial correlation estimation [1].

As large-scale event data are accumulated, studies on the construction of networks for events are becoming increasingly necessary. However, the occurrence of an event might often be only partially observed and finally recorded as censored. Censoring is a type of missing data problem commonly encountered in studies considering time-series data. Censoring can be attributed to various factors such as limitation of the study period and budget or abrupt discontinuation of the study. It representatively occurs in the form of left censoring, interval censoring, and right censoring, depending on the time of occurrence and observation of a censoring [7]. This study focuses on the right censoring problem, in which the censored time precedes the occurrence time for an event. Survival analysis provides a solution to various inference problems based on statistics, and the majority of studies have considered a single censored event with covariates [8]. However, subjects may experience multiple events within a study [9], and there may be a potential correlation structure between events despite the times to events being censored under independent distribution [10].

Several methods have been proposed to solve this problem in various fields and remarkably accurate estimations have often been achieved. However, there have been only a few appropriate solutions for estimating the partial correlation of times to the occurrence of multiple events, generally known as multivariate survival data. The censoring conceals the original times to the occurrences of events, which distorts the interrelations between the times and events. This problem is a considerable challenge because all of the times to events at each instance are not entirely observed owing to unknown reasons, that is, the survival times are right-censored. Essentially, there is difficulty in constructing the network for the variables of the censored data because of some missing values, and this difficulty is compounded by the multiple dimensionalities of the data.

Here, to solve this problem, multivariate survival analysis is considered to handle right-censored data. The distribution of right-censored variables is left-skewed because, in some samples, the value of the observed times is smaller than the true values. The censored data are calibrated based on bivariate nonparametric Bayesian estimates [11], yielding a probability density function for times to events. Using the probability density function, an empirical covariance matrix that guarantees non-negative definiteness is measured by the pairwise expectation on each variable. Then, Graphical Lasso is employed to select the non-zero entries of the inverse covariance matrix [6]. Using this procedure, the conditional dependencies are discovered, and the network that implies a partially correlated relationship between variables is constructed.

This paper proposes a method to detect pairs of partially correlated events for the multivariate survival data based on times to the occurrence of events. The remainder of this manuscript is organized as follows. In Section 2, the notation for the censored time-to-event data and the bases of approaches are briefly introduced, and the procedure of the proposed method is described. In Section 3, the performance of the proposed method as compared to competing methods for detecting target pairs is presented, based on numerical simulations performed in two types of network settings. Finally, in section 4, the results using the real dataset that consists of the first diagnosis of newborn babies for diseases are reported.

## Materials and methods

In this section, the proposed method for detecting the partially correlated events based on the times to events called censored network estimation (CNE) is described. In particular, it is considered that the times to events are censored independently of the true times. Essentially, CNE detects the partial correlation of the true times given the partially observed times rather than the true times. This method is based on the estimation for the joint probability density function for time-to-event data for multiple events and selection of non-zero partial correlation with inverse covariance estimation by lasso regression with vector-wise permutation. It reveals hidden relationships between events and visualizes the structure of events through an undirected network. The network consists of nodes indicating events and undirected edges indicating whether two nodes are partially correlated.

R source code for the proposed method is available at https://github.com/sunbisunbi/CNE.

### Multivariate survival data

This section describes the typical notation of survival analysis for information on the time taken for the event to occur and the censored time is considered for *N* samples and *J* events. In a typical case of multivariate survival analysis, *T*_*j*_ denotes the true occurrence time and *C*_*j*_ denotes the censored time for the event *j* = 1,2,3,…*j*. Let *T*_*j*_ = (*t*_1,*j*_,…,*t*_*n*,*j*_,…,*t*_*N*,*j*_) be a vector of the occurrence times where *t*_*n*,*j*_ is the true time-to-event of the event *j* for sample *n*. Let *C*_*j*_ = (c_1,*j*_,…,*c*_*n*,*j*_,…,*c*_*N*,*j*_) be a vector of the censored times, where *c*_*n*,*j*_ is the censored time of the event *j* for the sample *n*. Given *T*_*j*_ and *C*_*j*_, only (X_*j*_, Δ_*j*_) is observed, where *X*_*j*_ = (*x*_1,*j*_,…,*x*_*n*,*j*_,…,*x*_*N*,*j*_), Δ_*j*_ = (*δ*_1,*j*_,…,*δ*_*n*,*j*_,…,*δ*_*N*,*j*_), *x*_*n*,*j*_ = min (*t*_*n*,*j*_,*c*_*n*,*j*_) and δ_*n*,*j*_ = *I*(*t*_*n*,*j*_ ≤ *c*_*n*,*j*_) for *n* = 1,2,3,…*N*. Note that *T*_*j*_,*C*_*j*_,*X*_*j*_,Δ_*j*_ are vectors and *T*, *C*, *X*, Δ are matrices where *T* = (*T*_1_,…,*T*_*J*_), *C* = (*C*_1_,…,*C*_*J*_), *X* = (*X*_1_,…,*X*_*J*_), Δ = (Δ_1_,…,Δ_*J*_). That is, the event *j* has a set of observed times and censoring indicators represented as (*X*_*j*_,Δ_*j*_), or (X_*j*_ +) if Δ_*j*_ is zero, or (*X*_*j*_) if Δ_*j*_ is one [7]. Here, the representation of survival data (*X*_*j*_,Δ_*j*_) is used. Note that the true correlation of times to two events only depends on the occurrence times *T*, and the correlation cannot be completely measured because only *X* and Δ are given.

### Density estimation

As the covariance cannot be calculated if the true values are not given, it is difficult to directly estimate the partial correlation. To solve this problem, the multivariate survival analysis based on the optional Polya tree (OPT) Bayesian estimator [11] is applied here to estimate the joint probability density function of censored times to events. This enables the handling of bi-dimensional survival data.

Consider the calculation for the probability density with survival times. Let *f*_*i*,*j*_(*t*_*i*_,*t*_*j*_) be the joint probability density function of *T*_*i*_ and *T*_*j*_ for the true times to the occurrence of the events *i* and *j*, respectively. We estimate *f*_*i*,*j*_ by recursive binary splits of the bi-dimensional region through an optional Polya tree approach [12]. Let *A* be a region in the sample space, *A*_11_, *A*_12_ be regions partitioned by the *T*_*i*_-axis, and *A*_21_, *A*_22_ be regions partitioned by the *T*_*j*_-axis. Additionally, let Φ(*A*) be the likelihood for the region *A*, mathematically defined by

$$\phi (A)=\frac{1}{2}\phi_{0}(A)+\frac{1}{4}\sum_{m=1}^{2}\frac{B(N(A_{m}{}_{1})+0.5,N(A_{m}{}_{2})+0.5)}{B(0.5,0.5)}\phi (A_{m}{}_{1})\phi (A_{m}{}_{2}),$$

where B(·) is a beta function and Φ_0_(*A*) is the likelihood when all sample points are uniformly distributed, and *N*(*A*) is *NP*_*A*_, where *N* is the total number of observations and *P*_*A*_ is the probability mass obtained in region *A* by Kaplan-Meier’s survival estimator. If $\Phi_{0}\left(A\right)>\frac{1}{2}\sum_{m=1}^{2}\frac{B\left(N\left(A_{m1}\right)+0.5,\;N\left(A_{m2}\right)+0.5\right)}{B\left(0.5,\;0.5\right)}\Phi \left(A_{m1}\right)\Phi \left(A_{m2}\right)$, the probability density of *A* is calculated as $\frac{N\left(A\right)}{N\left|A\right|}$, where |*A*| is the area of *A*. If not, the probability density is considered to have a non-uniform distribution, and the region is split into the sub-region. If

$$\begin{array}{c} \frac{B(N(A_{11})+0.5,N(A_{12})+0.5)}{B(0.5,0.5)}\phi ({Α}_{11})\phi ({Α}_{12})\\ >\frac{B(N(A_{21})+0.5,N(A_{22})+0.5)}{B(0.5,0.5)}\phi ({Α}_{21})\phi ({Α}_{22}), \end{array}$$

*A* is split into *A*_11_ and *A*_12_. Otherwise, *A* is split into *A*_21_ and *A*_22_.

The estimator provides block-wise and uniformly distributed probability density based on a non-parametric Bayesian estimation. The details of this method are described in [11].

### Covariance matrix estimation

The covariance matrix of the times to multiple events can be obtained by the probability density function. Let *M* be the covariance matrix of the times to multiple events and *m*_*i*,*j*_ be the element of the matrix for entries *i* and *j* where *i* = 1,2,3,…,*J* and *j* = 1,2,3,…,*J*. If we know the joint probability density function *f*_*i*,*j*_ for times to two events, *m*_*i*,*j*_ is explicitly calculated as

$$m_{i,j}=\int_{t_{j}=0}^{\infty }\int_{t_{i}=0}^{\infty }\left(t_{i}-\int_{\tau_{i}=0}^{\infty }\tau_{i}\int_{\tau_{j}=0}^{\infty }f_{i,j}(\tau_{i},\tau_{j})d\tau_{i}d\tau_{j}\right)\left(t_{j}-\int_{\tau_{j}=0}^{\infty }\tau_{i}\int_{\tau_{i}=0}^{\infty }f_{i,j}(\tau_{i},\tau_{j})d\tau_{i}d\tau_{j}\right)f_{i,j}(t_{i},t_{j})dt_{i}dt_{j}.$$

Despite the clear and straightforward calculation, it is difficult to estimate the covariance given *X* and *Δ* and not *T*. Let ${\hat{f}}_{i,\;j}\left(t_{i},t_{j}\right)$ be the joint probability density function estimated by Bayesian estimator where (*X*_*i*_,*Δ*_*i*_) and (*X*_*j*_,*Δ*_*j*_) are given. The covariance matrix estimated by the above equation using ${\hat{f}}_{i,\;j}$ instead of *f*_*i*,*j*_ is not semi-positive definite because of the limitation of the probability estimation. The joint probability density function is estimated using only two events, without the conditional consideration of other events. The inconsideration causes inconsistencies in the probability density for events. The marginal probability density function of times to the event *i* can be approximated from ${\hat{f}}_{i,\;j}$,

$$f_{i}\left(t_{i}\right)\approx \int {\hat{f}}_{i,\;1}\left(t_{i},\;t_{1}\right)dt_{1}\approx \ldots \approx \int {\hat{f}}_{i,\;j}\left(t_{i},\;t_{j}\right)dt_{j}\approx \ldots \approx \int {\hat{f}}_{i,\;J}\left(t_{i},\;t_{J}\right)dt_{J}\;.$$

These derived marginal probability densities are similarly distributed but not identical. The marginal probability density is not consistently measured by estimating the joint probability density.

To avoid this effect, the covariance matrix is empirically calculated by estimating the true times to censored events rather than directly calculating from the joint probability density. The true times are estimated by the expectation of conditional probability for each sample. Clearly, if the time-to-event for a sample is not censored, the true time is equivalent to the observed time. However, when the time-to-event for a sample is censored, only the fact that the true time is larger than the observed time is given, and the true time is unknown. The true times of event occurrences are estimated by the expectation of the conditional probability given the observed times for the sample *n* and the event *j*,

$${\hat{t}}_{n,\;j}\;=\;E_{T_{j}}\left[T_{j}|x_{n,\;j},\;\delta_{n,\;j}\right]$$

$$=\;\left\{\begin{array}{c} x_{n,\;j}\;,\;\delta_{n,\;j}=1\\ E_{T_{j}}\left[T_{j}|T_{j}>x_{n,\;j}\right],\;\delta_{n,\;j}=0 \end{array}\right.\;.$$

Then, a set of the estimated times to the event are obtained as ${\hat{T}}_{j}=\left({\hat{t}}_{1,\;j},\;{\hat{t}}_{2,\;j},\;\ldots ,\;{\hat{t}}_{N,\;j}\right)'$. The expectation is approximated from a set of expectations for the marginal probability distribution derived from the joint conditional probability distribution for pairs of events. Let ${\hat{f}}_{j}^{k}$ be the estimated marginal probability density function for event *j* obtained from ${\hat{f}}_{j,\;k}$. Then, the expected time can be calculated as

$$E_{T_{j}}\left[T_{j}|T_{j}>x_{n,\;j}\right]≅\frac{1}{J}\sum_{k\;=\;1}^{J}E_{T_{j}\sim {\hat{f}}_{j,\;k}}\left[T_{j}|T_{j}>x_{n,j},x_{n,k},\delta_{n,k}\right]$$

$$=\;\frac{1}{J}\sum_{k\;=\;1}^{J}E_{T_{j}\sim {\hat{f}}_{j}^{k}}\left[T_{j}|{T_{j}>x}_{n,\;j}\right],$$

where ${\hat{f}}_{j}^{k}\left(t_{j}\right)=\int_{0}^{\infty }{\hat{f}}_{j,\;k}\left(t_{j},\;t_{k}\right)dt_{k}$. However, it is difficult to directly calculate the expectation of the conditional probability distribution. Therefore, the Monte Carlo simulation is used to approximate the above expectation. Following this, the covariance matrix is estimated by matrix multiplication of the estimated times and the average of the estimated times to each event as follows:

$$\hat{M}\;=\;\frac{1}{N}{\left(\hat{T}-1{\hat{\mu }}^{'}\right)}^{'}\left(\hat{T}-{1\hat{\mu }}^{'}\right),$$

where $\hat{\mu }\;=\;\left(\frac{1}{N}\sum_{n}{\hat{t}}_{n,\;1},\;\frac{1}{N}\sum_{n}{\hat{t}}_{n,\;2},\;\cdots ,\;\frac{1}{N}\sum_{n}{\hat{t}}_{n,\;J}\right)$ and **1** denotes a vector that consists of *N* elements of ones.

### Inverse covariance estimation

The typical algorithm for inverse covariance estimation, graphical lasso, is briefly described. Inverse covariance estimation was designed to detect the partial correlation of fully observed data. The algorithm is based on the maximization of *L*_1_-penalized Gaussian log-likelihood of the observations with respect to the mean parameter [5, 6]:

$$\log\;\det\;\Theta -tr\left(M\Theta \right)-\;\rho {\left\|\Theta \right\|}_{1},$$

where Θ is the inverse matrix of non-negative definite covariance. To solve this problem, the lasso regression was utilized. One column of the empirical covariance matrix of data is considered as response variables. And, columns excluding the column index corresponding to the response is considered as independent variables. It is iteratively calculated by permuting the target column and completed if it is converged.

For multivariate survival data, if all the times to events are fully observed, the above algorithm can estimate the inverse covariance matrix of the data. However, the covariance matrix of the times to events cannot be calculated completely because of the censoring. Censoring causes distortion of times to events and replaces the actual times of occurrence of events with censored times. Therefore, the log-likelihood of the estimated times to events is used here instead of the observations,

$$\log\;\det\;\Theta -tr\left(\hat{M}\Theta \right)-\;\rho {\left\|\Theta \right\|}_{1}.$$

The partial correlation of censored events is determined by the graphical lasso with the covariance matrix of the estimated times to events depending on the non-negative penalty ρ. If ρ is zero, all the absolute values of partial correlation between events will be greater than 0. As ρ increases from 0, less non-zero partial correlations are detected. Finally, the network based on non-zero partial correlation can be constructed for multiple censored events.

Additionally, the penalty parameter ρ can be selected through cross-validation to obtain a single network [13]. We briefly introduce the cross-validation for the graphical lasso. By partitioning the entire sample into *k*-fold, we can find an appropriate ρ that maximizes the summation of the log-likelihood of each fold. Let $M_{train}^{i}$ and $M_{valid}^{i}$ be the empicial covariance matrix of train set and validation set of fold *i*, respectively. Then, the log-likelihood can be calculated by

$$l_{i}\left(\rho \right)\;=\;-\log\;\det\;{\hat{\Sigma }}_{\rho }\left(M_{train}^{i}\right)-tr\left(M_{valid}^{i}{{\hat{\Sigma }}_{\rho }\left(M_{train}^{i}\right)}^{-1}\right),$$

where ${\hat{\Sigma }}_{\rho }\left(∙\right)$ denotes the covariance matrix estimated by the graphical lasso with ρ. Then, the penalty parameter is obtained by ${\hat{\rho }}_{CV}={argmax}_{\rho }k^{-1}\sum_{i=1}^{k}l_{i}\left(\rho \right)$. The details are described in [13]. The single network can be constructed through the above process.

### Data for the case study

The proposed method was applied in a case study to a real dataset to demonstrate its effectiveness. The *National Health Insurance Sharing Service (NHISS)* in South Korea has provided the *National Sample Cohort (NSC)*, which contains medical information of one million people extracted by random sampling from 2002 to 2015 for research purposes [14, 15]. For all samples, personal information, such as date of birth and place of residence, is masked to prevent identification of individuals. The *NCS* consists of about 2 billion medical events such as the date of diagnosis with disease codes, the month of death, and health screenings.

Here, the first diagnosis record for categorized disease codes was considered as an event of interest. Disease codes have been categorized via the *Korean Standard Classification of Diseases 7th Revision (KCD-7)* modified from the *International Statistical Classification of Diseases and Related Health Problems 10th Revision (ICD-10)* code [16].

The data can be found on the *NHISS* website (https://nhiss.nhis.or.kr). Details for the data used in the case study were described in S1 File. These data were accessed with *IRB-2018-0110* approval from *Korea University Institutional Review Board*. We declare no conflict of interest with the *NHISS*.

### Simulation studies

This section presents the performance of CNE recorded through simulation experiments. The main purpose of the simulation is to show the accuracy with which CNE detects non-zero partial correlations through the censored times to events. The simulation follows three steps:

1. Configure a network structure that represents the relation between events. Note that nodes and edges in the network represent events and non-zero partial correlations, respectively.
2. Generate the time-to-event data which follow the configured network and randomly censor occurrence times. The values of the data must be positive because they represent time, and the inverse covariance matrix of the data follows the configured network. Accordingly, the true times to events are not independent to each other. On the other hand, the censored time *C*_*j*_ is generated independently to *T*_*j*_ because the occurrence of censoring is irrelevant to the event of interest. It reflects that events can be censored by different reasons in real data and is a general way to simulate censoring in related works [11]. Note that the proposed method does not assume the independence of censoring.
3. Select the non-zero partial correlations of times to the occurrence of events given the survival data, *X*,*Δ*. The edge selection performance is evaluated from the area under the curve (AUC) and true positive rate (TPR) for false positive rates (FPRs) of 0.05 and 0.1 from the receiver operating characteristic (ROC) curve. The undirected edges that represent the true times being partially correlated are considered as conditional positives.

Following this, the survival data were generated based on the information of correlated events and censored times. Accordingly, the estimation of non-zero partial correlation was performed with the survival data. For all edges, the penalty parameter ρ_*j*_ was measured when the estimated absolute of the inverse covariance became greater than 0. The predicted positive was determined through ρ_*j*_. Three types of well-known networks, scale-free, random, small-world networks, were used in order to demonstrate that the proposed method is suitable for detecting partially correlated neighbors.

In addition, the proposed method was compared with the graphical lasso estimations based on the survival estimators of Dabrowska [17] and Lin-Ying [18]. Both are conventional approaches to estimating probability density functions for bivariate survival data. The upper bound performance was measured by the graphical lasso based on uncensored true times *T*. Additionally, the lower bound performance was measured based on the observed time *X* regardless of the censoring. The baseline was measured by the correlation coefficient of the observed times in uncensored samples, which have the censoring indicator of 1. The performance was investigated with repetitions and varied by changing the number of samples and events.

In addition to precedent simulation, the variation of performance was investigated by varying the censoring rate of events in each network. The simulation was performed repetitively, and the average, minimum, and maximum performances were specified.

### Simulation data generation

In this simulation, the detection performance was evaluated by comparing the edges estimated from time-to-event data with the true edges. To perform the simulation, the censored times to events were generated. The data have *N* samples and *J* events where *N* = 100,200,1000 and *J* = 20,50,100 and were generated through four steps.

First, an undirected network was generated and the network structure was considered. Edges of the network were considered as the conditional positive that should be detected, and nodes indicated events. The scale-free networks, random networks, and small-world networks were considered for this simulation.

Second, the true time matrix *T* was generated. The generation of the true times follows the simulation by Peng [1], but there is a difference in that all elements must be positive. The inverse covariance matrix should reflect the topology of the configured network. Essentially, the true time matrix *T* was determined by the undirected network structure. Let *P* denote the initial inverse covariance matrix and *P*(*i*,*j*) denote the element of *P* for entries of *i*,*j* = 1,…,*J*. Additionally, let Σ denote the covariance matrix of the true times and Σ(*i*,*j*) denote its element. To generate true times that reflect the network structure, *P* was set as follows:

$$P\left(i,j\right)\;=\;\left\{\begin{array}{c} 1,\;\;i=j;\\ 0,\;i\neq j,\;i≁j;\\ \;\tilde{\sigma },\;i\neq j,\;i\sim j, \end{array}\right.$$

where *i*~*j* and *i*≁*j* denote the existence and non-existence, respectively, of an edge between nodes *i* and *j*, and $\tilde{\sigma }$ is a correlation constant. In this simulation, $\tilde{\sigma }\;=\;0.8$ was used. To ensure the non-negative definiteness of the covariance matrix, *p*_*i*,*j*_ was rescaled by dividing itself by $2\times \left(\sum_{j=1}^{J}\left|p_{i,j}\right|-1\right)$ except for the diagonal element. Then, the rescaled matrix was symmetrized to be diagonally dominant. Let *A* be the symmetrized matrix and *A*(*i*,*j*) be its element. Then, the covariance matrix **Σ** which follows the preconfigured network is obtained by

$$\Sigma \left(i,j\right)\;=\;\frac{A^{-1}\left(i,\;j\right)}{\sqrt{A^{-1}\left(i,\;i\right)A^{-1}\left(j,\;j\right)}}\;.$$

For the specific case in which there are some isolated nodes in the network when $\sum_{j=1}^{J}\left|p_{i,j}\right|=1$, the rows and columns corresponding to indices of isolated nodes were first eliminated from the initial inverse covariance matrix. Following the above procedure, these rows and columns were inserted into the original position of Σ. Then, the non-negative true time-to-event data *T* were generated as the following log of the multivariate Gaussian distribution, where $\log\;\left(T\right)\sim N(0,\;\Sigma )$. Note that the true time-to-event data matrix *T* has *N* rows and *J* columns.

Third, the censored times *C* were generated. The censored times are assumed to be completely independent of true times and network structure. To reflect the irregular effect of censoring, *C*_*j*_ has no correlation with the corresponding true times and other censored times. The censored times were generated as following the exponential distribution:

$$C_{j}\sim \exp\;\left(\lambda \right),\;j\;=\;1,2,\cdots ,\;J,$$

where the mean of censored times to each event is $\frac{1}{\lambda }$. Different λ values were used for simulation.

Lastly, the survival data were composed. The observed time-to-event matrix *X* was obtained by min(*T*_*j*_,*C*_*j*_), and the censored time matrix Δ was obtained by *I*(*T*_*j*_ ≤ *C*_*j*_). The censored rate which represents the rate of censored samples can be represented by $1-\frac{1}{N}\sum_{n=1}^{N}\delta_{n,\;j}$; it changes based on the λ value. The censored rate moves closer to one if λ becomes larger.

### Simulation results

To show the proposed method is effective under diverse conditions, its performance when selecting partially correlated events was investigated. All simulation studies were performed under the scale-free, random, and small-world networks.

To provide evidence for the superiority of the proposed method, the selecting power of the upper bound, lower bound, baseline, and other competitive methods were investigated via simulation repetitions. Clearly, if the true times to events are provided, the partial correlation can be explicitly estimated by graphical lasso. Thus, the case when times are fully observed, i.e., *T* is given, was set as the ‘upper bound,’ while the case when the censored time *X* was provided without consideration of censoring was set as ‘lower bound.’ The case when estimating correlated edges by the absolute value of the correlation coefficient only by the samples in which both events are not censored was considered as ‘baseline.’ The estimators by Dabrowska and Lin and Ying were also adopted.

**Fig 1:**
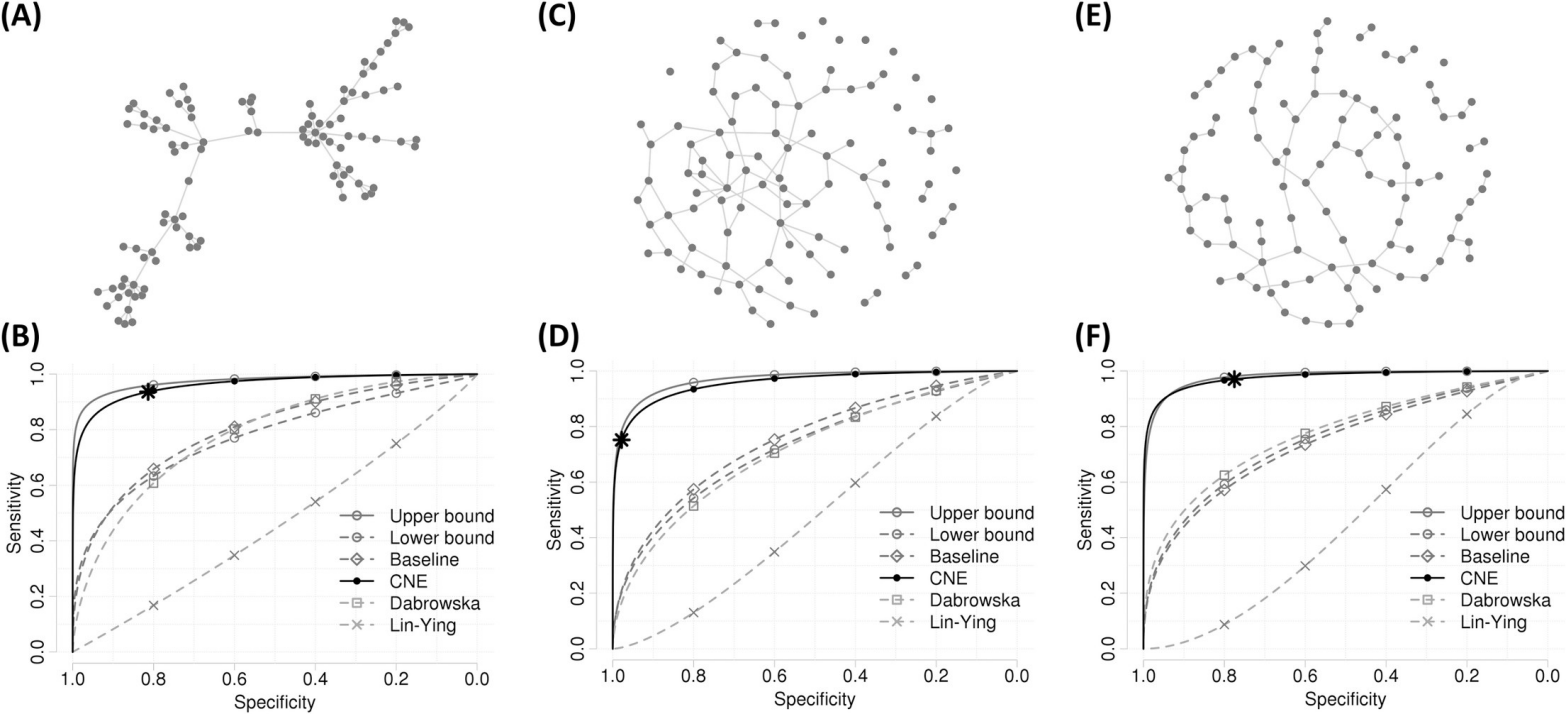
Topologies of the undirected network settings and the performance for detecting correlated events. ‘Upper bound’ denotes the case of given true time and ‘Lower bound’ denotes the network estimation using the censored times regardless of censoring. ‘Baseline’ denotes the selection by the absolute value of the correlation coefficient. ‘Dabrowska’ and ‘Lin-Ying’ indicate competing estimators for CNE. (A) Scale-free networks with 100 nodes and 99 edges. (B) Network estimation performance where the true times follow the network (A) and the censoring parameter **λ = 1**. (C) Random networks with 100 nodes and the probability of 0.02 to generate edge containing some isolated nodes. (D) Network estimation performance when the true times follow the network (C). (E) Small-world networks with 100 nodes and the rewiring probability of 0.15. (F) ROC curve for estimating the true edges of the network (E). * represents network estimation with cross-validation.

Fig 1 shows the simulation results for the network estimation in which there are 100 events corresponding to nodes and 1,000 samples for the events of interest. For generating the censored times, λ = 1 was used, and the censored rate ranged between about 50–70%. Fig 1A shows the scale-free network that has 99 edges corresponding to partially correlated pairs for the case in which some hub nodes of a comparatively larger degree exist. Fig 1B shows the performance of estimation for the scale-free networks. On average, the proposed method achieved an AUC of 0.96 with a TPR of 0.88 controlled at an FPR of 0.05, and a TPR of 0.92 controlled at an FPR of 0.1, with 10 repetitions. Additionally, the average AUCs of the upper bound, lower bound, baseline, Dabrowska estimator, and Lin-Ying estimator were 0.98, 0.8, 0.8, 0.81, and 0.46, respectively. An improvement of about 0.15 AUCs is shown in comparison to the lower bound, baseline, and competing method. The proposed method shows that the difference in performance is 0.02 AUCs in comparison to the upper bound despite the data being censored. The proposed method also shows stability with a standard error of about 0.01 for the AUCs. Fig 1C shows the random network, which has some isolated nodes and edges generated by the probability of 0.02. Fig 1D shows the performance of selecting a partial correlation for the random networks. On average, the proposed method also achieved a remarkable performance of 0.95 AUCs with a 0.81 TPR controlled at 0.05 FPR, and 0.87 TPR controlled at 0.1 FPR. Fig 1E shows the small-world network with the rewiring probability of 0.15. Fig 1F shows the ROC curve for network estimation for the small-world network in the presence of censoring. The proposed method achieved 0.98 AUCs with a 0.9 TPR controlled at 0.05 FPR, and 0.94 TPR controlled at 0.1 FPR. Additionally, networks estimated through cross-validation showed 0.94 TPR with 0.19 FPR, 0.75 TPR with 0.02 FPR, and 0.97 TPR with 0.22 FPR in Fig 1B, 1D and 1F, respectively. The cross-validation for the penalty parameter provided fairly dense networks in the scale-free and small-world network settings and sparse network in the random network setting. In all the methods except the Lin-Ying estimator, the network estimations are more effective in the scale-free and small-world networks than in random networks.

**Fig 2:**
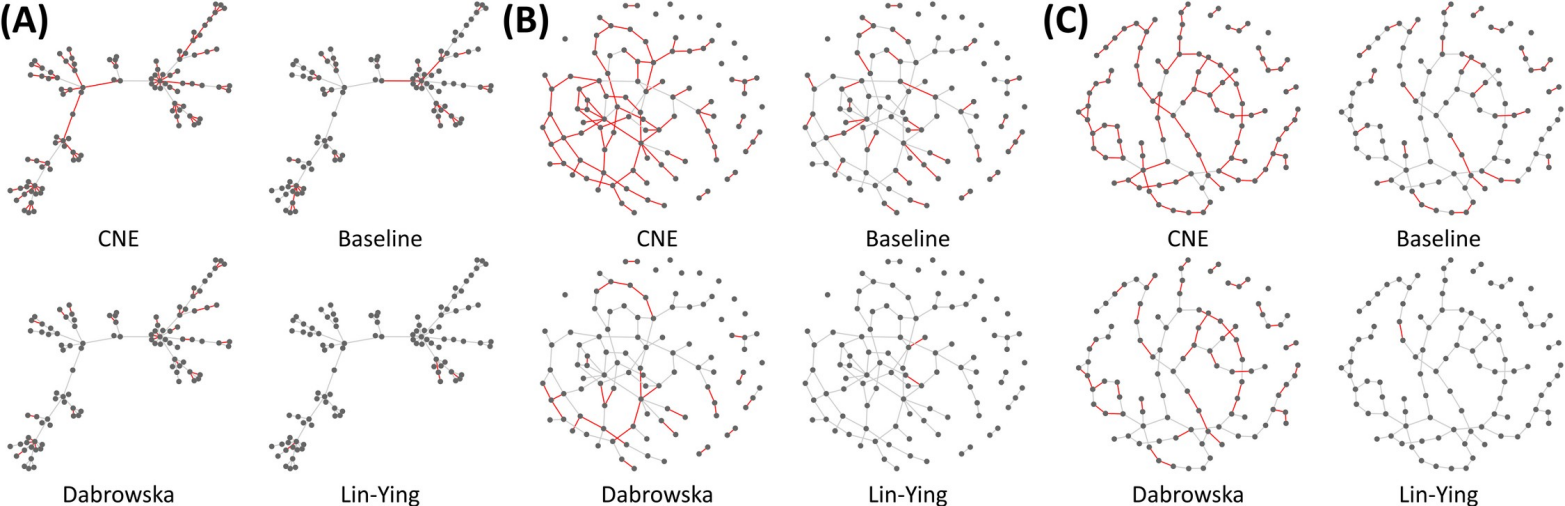
Edges detected by each estimator. (A) Scale-free network setting. (B) Random network setting. (C) Small-world network setting. Red lines indicate well-estimated edges controlled at FPR 0.05 in the network skeleton.

In addition to the above evaluation, we investigated which edges were found by estimators. In Fig 2, network skeletons correspond to networks in Fig 1A, 1C and 1E, respectively. Red lines indicate edges found by estimators and grey lines indicate that they were not found. The edge was selected at an FPR of 0.05. The proposed method located subsets that consist of edges connected to hub nodes more effectively than other estimators (Fig 2A). The proposed method also effectively found subsets that consisted of a few nodes and edges, as shown in Fig 2B. Fig 2C shows that the proposed method adequately estimated large clusters in the small-world setting.

To show the general case of network estimation, the methods were evaluated via ten repetitions by varying the simulation settings in the scale-free and random network settings (Table 1). The number of samples was considered as 100, 200, and 1000, while the number of events was considered as 20, 50, and 100, respectively. In this simulation, we set λ = 1, and both networks were used. In the scale-free network, there are *J* nodes whose degree distribution follows a power law and *J* − 1 edges. The random networks were generated with *J* nodes and the probability of 2/*J* that a node is connected to another node. The simulation results are shown in Table 1. We investigated the AUC and TPR at FPRs of 0.05 and 0.1 with respect to each method and summarized the averages and standard errors. The proposed method achieved the most powerful selection compared with baseline and competitive methods in all *N* and *J*. There is a tendency for the overall methods to show lower performance if the insufficient sample size is given, and the ratio of the dimension and sample number $\frac{J}{N}$ is low. In other words, the network estimation becomes challenging when N ≪ J [1] and it also affects the estimation of the probability density function with multivariate survival data [11]. In particular, the Lin-Ying estimator yields a negative probability density and leads to significantly lower performance.

**Table 1:** Summary of simulations with various sample numbers and event numbers. Each cell of the table consists of the average and standard error of AUC (top) and TPR at FPR of 0.05 (middle) and 0.1 (bottom) for simulations repeated ten times.

Network	*J*	*N*	Upper Bound	Lower Bound	Baseline	CNE	Dabrowska	Lin-Ying
Scale-free network	20	100	0.82 ± 0.06 0.28 ± 0.15 0.55 ± 0.13	0.57 ± 0.07 0.04 ± 0.03 0.11 ± 0.05	0.49 ± 0.09 0.06 ± 0.06 0.14 ± 0.09	**0.73 ± 0.05** **0.26 ± 0.16** **0.36 ± 0.18**	0.64 ± 0.07 0.08 ± 0.06 0.21 ± 0.1	0.46 ± 0.08 0.04 ± 0.04 0.05 ± 0.06
		200	0.89 ± 0.05 0.45 ± 0.2 0.69 ± 0.18	0.57 ± 0.08 0.07 ± 0.07 0.17 ± 0.06	0.63 ± 0.06 0.08 ± 0.04 0.21 ± 0.08	**0.79 ± 0.05** **0.38 ± 0.15** **0.51 ± 0.11**	0.68 ± 0.08 0.15 ± 0.08 0.25 ± 0.11	0.46 ± 0.05 0.02 ± 0.03 0.07 ± 0.05
		1000	0.97 ± 0.03 0.92 ± 0.1 0.96 ± 0.05	0.84 ± 0.05 0.48 ± 0.14 0.61 ± 0.11	0.82 ± 0.05 0.42 ± 0.12 0.56 ± 0.09	**0.96 ±** **0.03 0.88 ± 0.09** **0.93 ± 0.07**	0.88 ± 0.04 0.57 ± 0.13 0.69 ± 0.1	0.41 ± 0.05 0 ± 0 0.02 ± 0.03
	50	100	0.78 ± 0.03 0.28 ± 0.06 0.54 ± 0.08	0.53 ± 0.04 0.06 ± 0.03 0.13 ± 0.05	0.48 ± 0.04 0.06 ± 0.04 0.11 ± 0.05	**0.71 ± 0.03** **0.24 ± 0.06** **0.38 ± 0.08**	0.58 ± 0.04 0.09 ± 0.05 0.15 ± 0.04	0.47 ± 0.03 0.03 ± 0.03 0.07 ± 0.04
		200	0.9 ± 0.02 0.63 ± 0.04 0.81 ± 0.06	0.61 ± 0.03 0.13 ± 0.03 0.22 ± 0.04	0.64 ± 0.04 0.13 ± 0.05 0.24 ± 0.07	**0.83 ± 0.03** **0.5 ± 0.08** **0.63 ± 0.08**	0.67 ± 0.05 0.16 ± 0.04 0.3 ± 0.07	0.45 ± 0.04 0.03 ± 0.02 0.05 ± 0.02
		1000	0.97 ± 0.01 0.94 ± 0.03 0.96 ± 0.03	0.81 ± 0.04 0.44 ± 0.08 0.56 ± 0.05	0.8 ± 0.03 0.44 ± 0.08 0.56 ± 0.06	**0.96 ± 0.01** **0.86 ± 0.04** **0.92 ± 0.03**	0.87 ± 0.02 0.51 ± 0.08 0.68 ± 0.05	0.45 ± 0.03 0.02 ± 0.02 0.06 ± 0.03
	100	100	0.73 ± 0.02 0.29 ± 0.03 0.51 ± 0.04	0.52 ± 0.03 0.07 ± 0.02 0.14 ± 0.03	0.58 ± 0.02 0.07 ± 0.02 0.16 ± 0.04	**0.66 ± 0.02** **0.27 ± 0.05** **0.38 ± 0.06**	0.57 ± 0.02 0.09 ± 0.03 0.18 ± 0.03	0.48 ± 0.02 0.03 ± 0.02 0.07 ± 0.03
		200	0.86 ± 0.02 0.59 ± 0.07 0.76 ± 0.04	0.56 ± 0.03 0.1 ± 0.03 0.19 ± 0.03	0.61 ± 0.03 0.12 ± 0 03 0.21 ± 0.05	**0.81 ± 0.03** **0.46 ± 0.07** **0.6 ± 0.06**	0.62 ± 0.05 0.12 ± 0.04 0.25 ± 0.06	0.46 ± 0.03 0.02 ± 0.02 0.06 ± 0.02
		1000	0.98 ± 0.01 0.94 ± 0.02 0.96 ± 0.02	0.8 ± 0.02 0.45 ± 0.07 0.56 ± 0.05	0.8 ± 0.02 0.41 ± 0.06 0.53 ± 0.04	**0.96 ± 0.01** **0.88 ± 0.02** **0.92 ± 0.02**	0.81 ± 0.03 0.41 ± 0.07 0.59 ± 0.05	0.46 ± 0.02 0.02 ± 0.01 0.05 ± 0.02
Random network	20	100	0.77 ± 0.06 0.25 ± 0.16 0.44 ± 0.16	0.56 ± 0.08 0.08 ± 0.07 0.13 ± 0.1	0.5 ± 0.08 0.06 ± 0.05 0.13 ± 0.1	**0.67 ± 0.07** **0.2 ± 0.08** **0.32 ± 0.1**	0.66 ± 0.05 0.19 ± 0.12 0.27 ± 0.15	0.44 ± 0.06 0.04 ± 0.05 0.06 ± 0.07
		200	0.93 ± 0.05 0.75 ± 0.2 0.89 ± 0.15	0.61 ± 0.13 0.13 ± 0.1 0.21 ± 0.16	0.65 ± 0.11 0.14 ± 0.11 0.2 ± 0.12	**0.88 ± 0.08** **0.64 ± 0.18** **0.73 ± 0.14**	0.79 ± 0.09 0.22 ± 0.17 0.36 ± 0.2	0.46 ± 0.08 0.03 ± 0.04 0.04 ± 0.05
		1000	0.96 ± 0.04 0.88 ± 0.11 0.94 ± 0.08	0.74 ± 0.06 0.27 ± 0.15 0.37 ± 0.14	0.69 ± 0.06 0.24 ± 0.12 0.38 ± 0.13	**0.91 ± 0.05** **0.73 ± 0.12** **0.8 ± 0.08**	0.8 ± 0.05 0.33 ± 0.18 0.51 ± 0.13	0.48 ± 0.08 0.01 ± 0.02 0.06 ± 0.06
	50	100	0.72 ± 0.04 0.22 ± 0.08 0.42 ± 0.07	0.52 ± 0.02 0.06 ± 0.03 0.12 ± 0.04	0.49 ± 0.06 0.06 ± 0.04 0.11 ± 0.05	**0.65 ± 0.05** **0.2 ± 0.07** **0.3 ± 0.08**	0.55 ± 0.04 0.08 ± 0.04 0.15 ± 0.07	0.47 ± 0.04 0.02 ± 0.02 0.06 ± 0.03
		200	0.84 ± 0.04 0.4 ± 0.1 0.61 ± 0.08	0.58 ± 0.03 0.08 ± 0.05 0.18 ± 0.04	0.61 ± 0.03 0.1 ± 0.06 0.19 ± 0.08	**0.76 ± 0.06** **0.34 ± 0.07** **0.48 ± 0.09**	0.63 ± 0.06 0.14 ± 0.05 0.23 ± 0.07	0.48 ± 0.03 0.03 ± 0.02 0.06 ± 0.03
		1000	0.97 ± 0.01 0.91 ± 0.05 0.95 ± 0.03	0.77 ± 0.05 0.37 ± 0.11 0.5 ± 0.1	0.74 ± 0.03 0.34 ± 0.08 0.46 ± 0.05	**0.95 ± 0.02** **0.8 ± 0.06** **0.88 ± 0.05**	0.84 ± 0.03 0.4 ± 0.06 0.56 ± 0.07	0.46 ± 0.04 0.03 ± 0.02 0.05 ± 0.03
	100	100	0.66 ± 0.04 0.2 ± 0.07 0.37 ± 0.07	0.51 ± 0.03 0.06 ± 0.01 0.12 ± 0.03	0.51 ± 0.04 0.06 ± 0.02 0.13 ± 0.03	**0.6 ± 0.04** **0.17 ± 0.03** **0.28 ± 0.04**	0.53 ± 0.03 0.06 ± 0.02 0.15 ± 0.04	0.48 ± 0.02 0.04 ± 0.02 0.08 ± 0.02
		200	0.8 ± 0.03 0.41 ± 0.06 0.58 ± 0.06	0.57 ± 0.03 0.1 ± 0.03 0.19 ± 0.03	0.58 ± 0.02 0.09 ± 0.04 0.18 ± 0.04	**0.72 ± 0.05** **0.31 ± 0.07** **0.42 ± 0.08**	0.6 ± 0.04 0.1 ± 0.03 0.2 ± 0.05	0.48 ± 0.02 0.04 ± 0.01 0.06 ± 0.02
		1000	0.97 ± 0.01 0.92 ± 0.06 0.95 ± 0.04	0.73 ± 0.05 0.3 ± 0.07 0.42 ± 0.09	0.72 ± 0.04 0.29 ± 0.05 0.41 ± 0.05	**0.95 ± 0.03** **0.81 ± 0.07** **0.87 ± 0.06**	0.74 ± 0.05 0.33 ± 0.06 0.48 ± 0.08	0.47 ± 0.03 0.02 ± 0.02 0.05 ± 0.02

The above simulation was carried out on 24 CPU cores, which are Intel (R) Xeon (R) E5-2630 v2 @ 2.60GHz and 128GB RAM. The average computation time of each simulation is summarized in Table 2.

**Table 2:** Average computation time (second) of censored network estimation for three types of networks according to the number of nodes and samples.

*J* *N*	20	50	100
100	192	1,295	4,296
200	346	2,519	8,931
1,000	1,558	12,369	63,936

**Fig 3:**
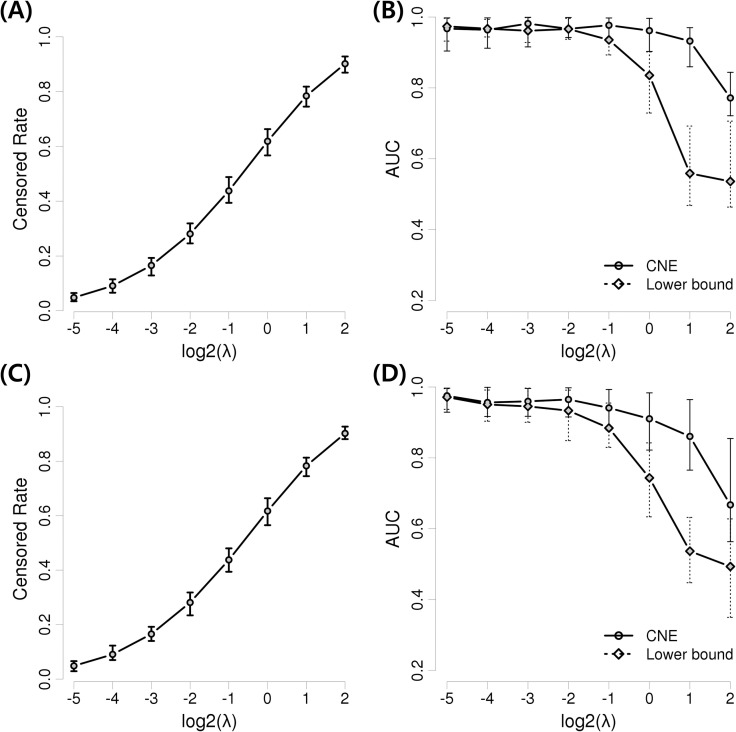
Censored rate and AUC for the change of censored time parameter λ. (A) Variation of the censored rate in the scale-free network simulation. (B) Variation of AUC in the scale-free network simulation. (C) Variation of the censored rate in the random network. (D) Variation of AUC in the random network. Points represent the average values, upper bars represent the maxima, and lower bars represent the minima.

In addition, the selection power of the proposed method and the lower bound were investigated by changing the parameters for generating the censored times with respect to 1,000 samples and 20 events. The censored times were generated based on the exponential distribution with parameter λ. If λ changes, the censored rate of an event also changes. We took λ = 2^−5^, 2^−4^,…, 2^2^, and the simulation was repeated ten times per λ value. Fig 3A and 3C show the ranges of the censored rates for the scale-free and random networks. When the expectations of the true and censored times are identical, the censored rate was about 50–70%. Fig 3B and 3D show changes in the AUCs for the network estimation based on λ in the scale-free and random networks. The AUCs of both methods were over 0.9 up to λ = 2^−2^. From λ = 2^−1^, performance differences begin to emerge. When λ = 2^0^, the AUCs of the proposed method and lower bound were 0.96 and 0.84, respectively. When λ = 2^1^, 2^2^, the lower bound performance was under 0.5, which is as weak as random selection. In contrast, the AUCs of the proposed method were 0.72–0.97 (B) and 0.56–0.96 (D) for the scale-free and random networks, respectively, despite the censored rate being 75–93%.

**Fig 4:**
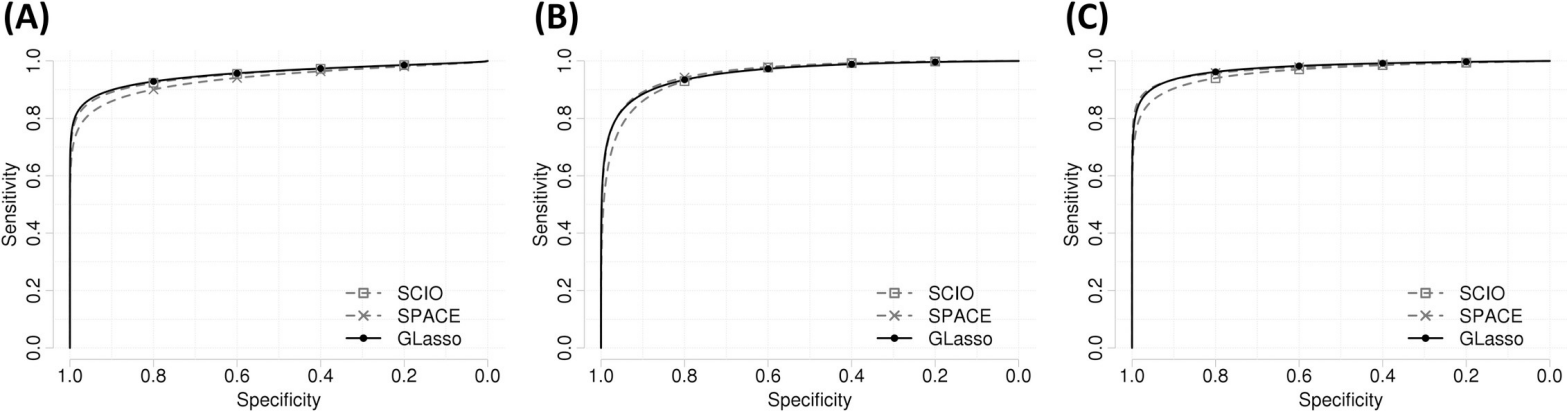
Comparison of network estimation methods after the covariance matrix measure. (A) Scale-free network. (B) Random network. (C) Small-world network.

Lastly, we investigated the performance of three network estimation methods with the covariance matrix measured from multivariate censored data with 1,000 samples and 100 events. The compared methods are sparse columnwise inverse operator (SCIO) [19], sparse partial correlation estimation with degree-based weights (SPACE) [1], and graphical lasso (GLasso). Fig 4 shows the ROC curves for the network estimation in each network setting. AUCs were evaluated as 0.942, 0.921, 0.946 in the scale-free network setting, 0.95, 0.961, 0.96 in the random network setting, and 0.963, 0.974, 0.977 in the small-world setting (SCIO, SPACE, GLasso). There were slight performance gap between the three methods, but the methods showed similar estimation performance.

### Application studies

The proposed method was applied to a case study to investigate the relationship with the next disease after the initial diagnosis of ‘acute upper respiratory infections’ for the newborn baby dataset. It is important to detect connections to other diseases because the ‘acute upper respiratory infections’ are a common disease experienced by many newborns [20]; in the dataset, 99.49% of newborns had been diagnosed with them. Additionally, the number of samples is sufficient to apply the method.

The *NCS* contains a table composed of the diagnostic date and primary sick symbol for one million samples. Among all the samples, we focused on a dataset of newborns born between 2002–2006 to identify the date of the first diagnosis. In particular, 13,735 babies who were first diagnosed with the ‘acute upper respiratory infections’ were selected to explicitly identify time-to-event data. Accordingly, the time of the dataset was measured by a period from the ‘acute upper respiratory infections’ to diagnoses for other diseases. The dates of the first diagnosis were collected by tracking the medical records of the babies up to 2015. If there is no record for the diagnosis of a categorized disease until 2015, the event is considered as censored. Additionally, only 36 disease categories with observation rates exceeding ten percent were considered instead of the entire disease data because disease categories with low observation rates were rarely connected to the others. The disease code, title of the disease, and censored rates of each of the diseases are listed in Table 3. ‘Other acute lower respiratory infections’ were the most observed and ‘disorders of skin appendages’ were the least observed cases in the dataset.

**Table 3:** Summary of disease codes with an observation rate of over 10% for newborn babies who were first diagnosed for acute upper respiratory infections.

Disease Code	Title	Obs. Rate (%)	Disease Code	Title	Obs. Rate (%)
A00-A09	Intestinal infectious diseases	87.8	L00-L08	Infections of the skin and subcutaneous tissue	62.5
B00-B09	Viral infections characterized by skin and mucous membrane lesions	49.3	L20-L30	Dermatitis and eczema	86.6
B25-B34	Other viral diseases	20.1	L50-L54	Urticaria and erythema	49
B35-B49	Mycoses	16.6	L60-L75	Disorders of skin appendages	10.2
H00-H06	Disorders of eyelid, lacrimal system and orbit	42.8	L80-L99	Other disorders of the skin and subcutaneous tissue	15.2
H10-H13	Disorders of conjunctiva	75.2	M00-M25	Arthropathies	13.3
H15-H22	Disorders of sclera, cornea, iris and ciliary body	18	M60-M79	Soft tissue disorders	27
H49-H52	Disorders of ocular muscles, binocular movement, accommodation and refraction	62.3	N30-N39	Other diseases of the urinary system	21.1
H60-H62	Diseases of external ear	39.3	R00-R09	Symptoms and signs involving the circulatory and respiratory systems	20.9
H65-H75	Diseases of middle ear and mastoid	82.3	R10-R19	Symptoms and signs involving the digestive system and abdomen	41.4
J09-J18	Influenza and pneumonia	59.0	R50-R69	General symptoms and signs	41.4
J20-J22	Other acute lower respiratory infections	99	S00-S09	Injuries to the head	54
J30-J39	Other diseases of upper respiratory tract	91.8	S50-S59	Injuries to the elbow and forearm	25.4
J40-J47	Chronic lower respiratory diseases	76.4	S60-S69	Injuries to the wrist and hand	46.3
K00-K14	Diseases of oral cavity, salivary glands and jaws	23	S80-S89	Injuries to the knee and lower leg	25.8
K20-K31	Diseases of oesophagus, stomach and duodenum	49.8	S90-S99	Injuries to the ankle and foot	51
K50-K52	Noninfective enteritis and colitis	47.9	T15-T19	Effects of foreign body entering through natural orifice	16.3
K55-K64	Other diseases of intestines	53.2	T20-T32	Burns and corrosions	18.2

Using the proposed method, the correlations between diseases were analyzed based on the times to the first diagnosis. The intensively correlated diseases were estimated by cutting the top five percent partial correlation among all possible pairs. The interrelation was visualized as an undirected network. The relation and each disease were represented by edges and nodes, respectively. In this network, the size of each node indicates its degree, and three of the most highly correlated edges have been marked with a bold line.

**Fig 5:**
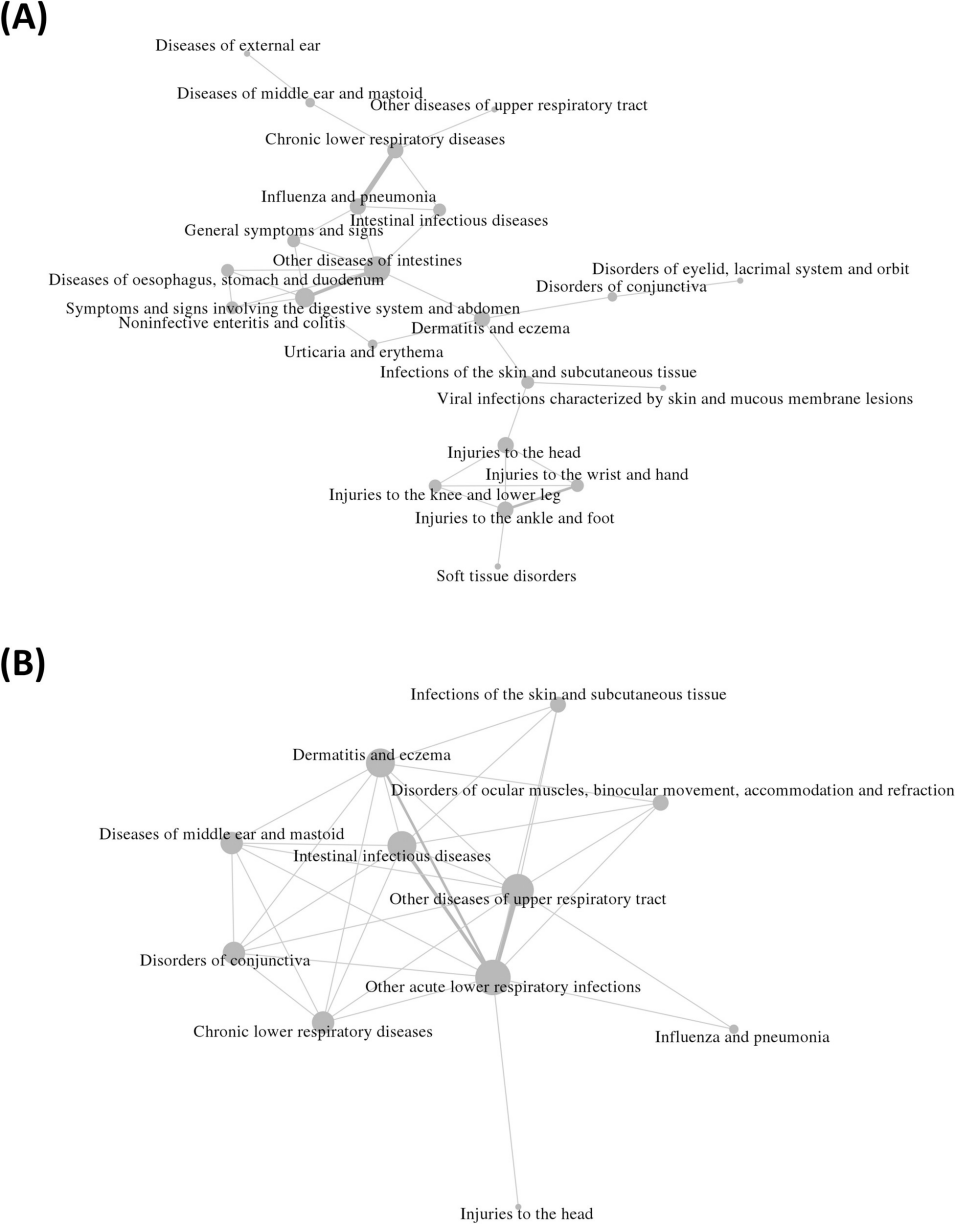
Disease networks estimated from the first diagnosis of the newborn baby dataset in Korea. (A) Correlation network estimated by CNE. (B) Co-occurrence network within the observation period.

Fig 5A shows the correlation network estimated by the proposed method based on times to the first diagnoses of diseases after ‘acute upper respiratory infections’ in babies. To compare the results, another disease pair was estimated by the co-occurrence of diseases within the observation period [21], as shown in Fig 5B. The co-occurrence network indicates that the pair of diseases were frequently observed within the study period. On the other hand, the correlation network represents an analytical relation for the diagnostic time points.

Using CNE and co-occurrence, 32 pairs of interrelated diseases were found within 36 disease codes. For example, three of the most intensive correlations, (‘influenza and pneumonia,’ ‘chronic lower respiratory diseases’), (‘other intestinal diseases,’ ‘symptoms and signs involving the digestive system and abdomen’), and (‘injuries to the wrist and hand,’ ‘injuries to the ankle and foot’), were found in the correlation network. The most intensive correlation has been explicitly demonstrated through research on the effect of influenza and pneumonia on lower respiratory infection [22, 23]. The second highly correlated pair corresponds to symptoms of the digestive system due to intestinal problems, leading to hospital visits [24]. The third intensive pair can be explained by the association of injuries to the wrist and hand and injuries to the ankle and foot [25]. The correlation tends to be affected by times to events rather than the observation rate of each disease. The above example is clearly a plausible case based on advanced research. In contrast, the above example could not be found in the conventional co-occurrence network; instead, (‘other acute lower respiratory infections,’ ‘other diseases of upper respiratory tract’), (‘other acute lower respiratory infections,’ ‘intestinal infectious diseases’), and (‘other acute lower respiratory infections’, ‘dermatitis and eczema’) were representatively found. The first case is marginally reliable, and the third case could be supported by the conventional concept of inclusion of the lower respiratory illness in atopic diseases in infants [26], whereas the second case was difficult to demonstrate based on existing clinical research. In addition to the example, there are several improbable cases in the co-occurrence network, such as (‘disorders of conjunctiva,’ ‘chronic lower respiratory diseases’) and (‘intestinal infectious diseases,’ ‘disorders of ocular muscles, binocular movement, accommodation and refraction’). Additionally, the co-occurrence network showed that the co-occurrence tends to be strictly dependent to the observation rate rather than times to events. This implies that the correlation network based on times to events is more appropriate to reveal a potential relationship between diseases. Therefore, we suggest a researchable hypothesis for a new discovery for probable relationships of diseases in the correlation network.

This work was carried out on the remote windows server of Intel(R) Xeon(R) CPU E5-2690 v4 A 2.60GHz and 3.00GB RAM provided by *NHISS*. The computation time was 73,170 seconds for 13,735 samples and 36 events.

## Discussion

In this paper, an extended approach to detect the non-zero partial correlation of multivariate survival data in the presence of censoring is presented. The proposed method is based on the graphical lasso and multivariate survival analysis and determines the non-zero partial correlation by a certain threshold of the L1 penalty. This method achieved remarkable results in simulation studies compared to three competing methods, even demonstrating performance close to the analysis when given all true times. In the application to real datasets, the method provided an interpretable correlation between categorized diseases through related medical literature.

We dealt with the network estimation from censored time-to-event data for multiple events. This work can be extended to other contexts in terms of density estimation for multivariate analysis. Simolo et al. suggested missing value estimation by density function for precipitation [27]. It can be compared with our covariance matrix measure and extended to network estimation from missing variables. It implies that the network estimation problem can be considered not only for censored data.

During the implementation of the proposed method, the probability distributions of all pairs were estimated. Performing all the calculations can be computationally inefficient and may not be necessary. To improve this, a gradual convergence approach beginning from the analysis of the data as it is censored or parallel computation can be considered.

The proposed method was demonstrated to be capable of obtaining reliable interrelations beyond assumptions that do not reflect the real world in conventional survival analysis. This work might be able to cover the network reconstruction problem in different domains if a single variable holds the fitness of Kaplan-Meier estimation. For example, the relations between multiple censored factors on astronomical data [10] could be inferred, or the proposed method can provide an opportunity of multidirectional reliability analysis for manufacturing systems [28]. The method is expected to be applicable to uncover interrelations between censored events in various fields.

## Supporting information

S1 File (DOCX)
