# Supplementary material for: Network estimation for censored time-to-event data for multiple events based on multivariate survival analysis
Source: PLoS One. 2020 Oct 1;15(10):e0239760. doi: 10.1371/journal.pone.0239760 (PMC7529251; doi:10.1371/journal.pone.0239760)
Supplement: S1 File — (DOCX) [file pone.0239760.s001.docx]

Network estimation for censored time-to-event data for multiple events based on multivariate survival analysis

Yoojoong Kim^1^, Junhee Seok^1*^

^1^School of Electrical Engineering, Korea University, Seoul, South Korea.

**Supplementary Methods**

**Data Source**

Here we describe the National Sample Cohort (NSC) data [1, 2] used in the application studies of this paper and its application. The data were provided by the National Health Insurance Sharing Service (NHISS) in South Korea (https://nhiss.nhis.or.kr). On the website, the NSC data is named as ‘Sample cohort DB’ and can be found in the ‘Sample Research DB’ menu. It consists of a million samples that were randomly extracted from the whole national health insurance subscribers and four types of databases that are Qualification DB, Treatment DB, Medical check-up DB, and Clinic DB. In this work, Qualification DB and Treatment DB among four databases were used.

Note that we are not allowed to share the data by ourselves by the regulation of the NHISS. The data can be only accessed through the NHISS. To access these databases, the study application form with an IRB approval should be submitted to the NHISS. Once the study is approved, users can handle the databases via a cloud server of the NHISS. Raw data is not able to download due to the policy of the NHISS. All analyses in the application studies need to be carried out upon the cloud server.

Qualification DB contains a single table, and it consists of 14 variables that indicate information on subjects such as patient id, gender, age group, etc. The patient id is a unique index and matches the identical subject on other databases. Also, we could identify the newborn baby sample through age group. In addition, the death date can be inquired from Qualification DB. We could check censored times of events by death date.

Treatment DB includes ten types of tables. Among them, we focused on ‘Type of disease table (40t)’ for the medical institution that contains patient id, the start date of medical care, and the main sick. The main sick was grouped into disease groups by following the ICD-10 code. Each disease group was considered as an event. Then, the first time to medical care for each event was gathered, and it was considered as times to events.

## Reference

1. Lee J, Lee JS, Park S-H, Shin SA, Kim K. Cohort profile: The national health insurance service–national sample cohort (NHIS-NSC), South Korea. Int J Epidemiol. 2016;46(2):e15-e.

2. Seong SC, Kim Y-Y, Park SK, Khang YH, Kim HC, Park JH, et al. Cohort profile: The national health insurance service-national health screening cohort (NHIS-HEALS) in Korea. BMJ Open. 2017;7(9):e016640.
